# Skin Manifestations after Ionizing Radiation Exposure: A Systematic Review

**DOI:** 10.3390/bioengineering8110153

**Published:** 2021-10-22

**Authors:** Luigi Bennardo, Maria Passante, Norma Cameli, Antonio Cristaudo, Cataldo Patruno, Steven Paul Nisticò, Martina Silvestri

**Affiliations:** 1 Department of Health Science, Magna Graecia University, 88100 Catanzaro, Italy; maria.passante@studenti.unicz.it (M.P.); cataldopatruno@libero.it (C.P.); nistico@unicz.it (S.P.N.); martinaeg@hotmail.it (M.S.); 2 Istituto Dermatologico San Gallicano IRCSS, IFO, 00100 Rome, Italy; norma.cameli@ifo.gov.it (N.C.); antonio.cristaudo@ifo.gov.it (A.C.)

**Keywords:** radiation exposure, skin manifestations, radiation dermatitis

## Abstract

Morphological and functional skin alterations secondary to the action of ionizing radiation are well documented. In addition to its application in the medical field, ionizing radiation represents a public health problem for diagnostic and therapeutic purposes due to the potential risk of exposure to unexpected events, such as nuclear accidents or malicious acts. With regard to the use of ionizing radiations in the medical field, today, they constitute a fundamental therapeutic method for various neoplastic pathologies. Therefore, the onset of adverse skin events induced by radiation represents a widespread and not negligible problem, affecting 95% of patients undergoing radiotherapy. A systematic literature search was performed from July 2021 up to August 2021 using PubMed, Embase, and Cochrane databases. Articles were screened by title, abstract and full text as needed. A manual search among the references of the included papers was also performed. This systematic review describes the various skin reactions that can arise following exposure to ionizing radiation and which significantly impact the quality of life, especially in cancer patients.

## 1. Introduction

Ionizing radiation is capable of causing morphological and functional alterations in the tissues. In addition to being an important treatment modality for cancer patients, ionizing radiation is also a public health concern due to the potential for nuclear and radiological events [1]. Fluoroscopic imaging is one of the primary sources of exposure to ionizing radiation in the medical field; fluoroscopy uses X-rays to obtain dynamic anatomical images in real-time and is used in many specialties, including interventional radiology, interventional cardiology, orthopedics, urology, vascular surgery, and gastroenterology [2].

The extent of radiation damage is related to several factors, including the total radiation dose, tissue irradiation volume, and the radiation dose’s time interval [3]. The highly proliferative and oxygenated cells are the most sensitive to radiation damage; therefore, the most radiosensitive organs are the bone marrow, the reproductive and gastrointestinal systems, the skin, the muscles, and the brain. The skin is a continuously renewing organ composed of rapidly proliferating cells and is susceptible to ionizing radiation. The most radiosensitive cells of the integumentary system are the basal keratinocytes, stem cells of the hair follicle, and melanocytes [4]. When the skin undergoes the action of ionizing radiation, a percentage of basal keratinocytes are destroyed by the first dose of radiation, disrupting the skin’s ability to self-renew; in addition to the basal keratinocytes, the stem cells of the hair follicle are also injured, due to damage to nuclear and mitochondrial DNA induced by free radicals [5]. Damage to the basal keratinocytes induces the release of various cytokines and chemokines and stimulates Langerhans cells, dendritic cells, mast cells, T cells, neutrophils, and macrophages [6,7].

Radiation-induced skin damage occurs in approximately 95% of patients undergoing cancer radiotherapy and has also been reported in over 70 fluoroscopic procedures [8]. Radiation skin damage can be divided into acute or late (chronic); acute damage occurs within hours or weeks of radiation exposure, while chronic damage occurs months or years after radiation exposure [1]. Several factors influence the development of radiation skin damage, including treatment-related and patient-related factors.

Treatment-related factors include radiation dose, irradiated anatomical area, fractionation and exposure times, and radiation beam angle. The skin areas most sensitive to radiation are the anterior region of the neck, chest, extremities, abdomen, and face. Individual predisposing factors include sex, age, obesity, chronic ultraviolet exposure, smoking, and various medical conditions, including Gorlin Syndrome, ataxia telangiectasia, connective tissue disorders, xeroderma pigmentosum, Gardner’s syndrome, hereditary malignant melanoma, and dysplastic nevus syndrome. The role of atopic dermatitis in the development of radiation skin injuries is debated [9].

## 2. Materials and Methods

A comprehensive literature search to identify relevant studies was carried from July 2021 up to August 2021, with no temporal restriction, using the following databases: MEDLINE/PubMed (National Center for Biotechnology Information, NCBI), EMBASE (Ovid), and the Cochrane Central Register of Controlled Trials (CENTRAL). The search string contained Medical Subject Headings (MeSH) and free-text terms.

The following key search terms have been used in order to include the maximum number of relevant papers: “Cutaneous adverse events”, “Radiations”, “Radiotherapy/adverse effects”, “Dermatitis, Radiation-Induced” and “Neoplasms, Radiation-Induced”.

In addition, a search for citations in the reference list of the selected articles was conducted. The Preferred Reporting Items for Systematic Reviews and Meta-Analyses (PRISMA) methodology selected studies based on the following criteria. Only studies that met the following inclusion criteria were included: (a) papers in the English language, (b) all cited studies must have been approved by an ethics committee or an institutional review committee. Exclusion criteria were: (a) papers that are not on-topic (b) studies that do not provide sufficient data on the topic. Reviews, observational studies, case series, and case reports were included. No temporal restrictions were applied. After eliminating the duplicates, the eligible articles were screened based on the title and abstract; finally, the full text of the articles potentially suitable for inclusion in the systematic reviews was analyzed.

## 3. Results

**Figure 1:**
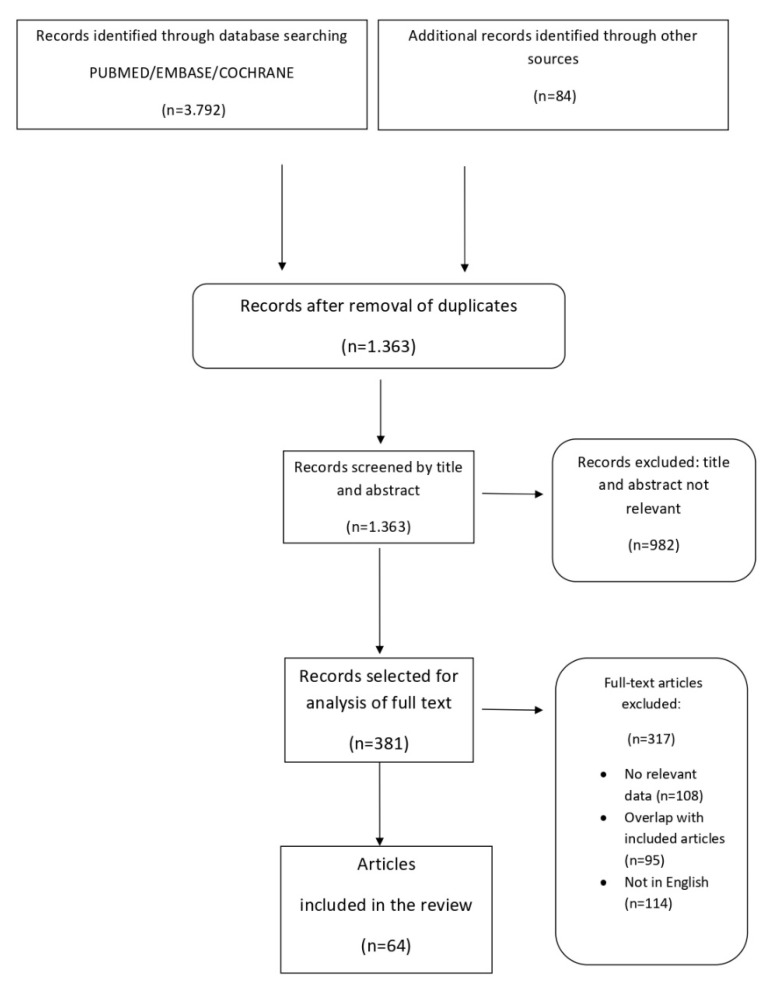
Article selection flowchart.

The search identified 3792 references, and the screening of titles and abstracts has removed 982 references. The full text of the remaining 381 papers has been assessed for inclusion; finally, 64 papers met the inclusion criteria, therefore, have been included in the review. The article selection flow chart (see Figure 1) summarizes the search strategy adopted in this study.

Several radiation-induced skin complications have been reported in the literature, including radiation dermatitis, radiation recall dermatitis, radiation-induced skin malignancies, radiation-induced morphea, radiation-induced bullous pemphigoid, lymphangioma circumscriptum, pseudosclerodermatous panniculitis, radiation port dermatophytosis, lichen planus, Langheran cell histiocytosis, systemic mastocytosis, radiation port erythema multiforme, solitary xanthogranuloma.

## 4. Discussion

### 4.1. Radiation Dermatitis

Radiation dermatitis is one of the main side effects of radiation exposure and affects 95% of patients undergoing radiotherapy [10]. Acute radiation dermatitis occurs within 90 days of exposure to ionizing radiations, while the chronic form may develop months or years after treatment. Radiation dermatitis is associated with a radiation dosage of 2–50 gray [11].

The first dose of radiation causes acute damage and destruction of the basal keratinocytes; with the persistence of exposure to ionizing radiation, the process continues, thus determining structural and histological changes in the skin and connective tissue [12]. Radiation-induced damage is mediated by free radicals that damage DNA, proteins, lipids, and carbohydrates. Impairment of stem and endothelial cells, recruitment of inflammatory cells, apoptosis, and necrosis of epidermal cells follow from acute damage [13,14]. Clinical manifestations of acute radiation dermatitis vary with the amount of radiation exposure and range from erythema to desquamation and ulceration; the National Cancer Institute has ranked the severity of the acute disease on a scale of 1 to 4 [15].

Mild erythema caused by dilation of the capillaries may occur within hours of the patient’s exposure to radiation; erythema can be associated with edema, itching, and burning. In addition, hair follicles and sebaceous glands can be affected, resulting in skin xerosis and hair loss. Dry desquamation may occur at cumulative doses above 20 Gy, usually after 3–6 weeks of radiation treatment. Wet desquamation occurs when radiation doses exceed 30–40 Gy and is characterized by serous exudate and potential development of bullous lesions [16]; at this stage, the risk of infections and ulcer formation increases. In severe desquamation, it is advisable to suspend the radiotherapy treatment to allow the re-epithelialization processes. Acute skin manifestations are generally evident 1–2 weeks after completion of radiotherapy and resolve 2 to 4 weeks after the end of treatment; however, it is possible the persistence of post-inflammatory hyperpigmentation, which reduces over time [17].

Multiple data suggest that an imbalance of pro-inflammatory and profibrotic cytokines is involved in the pathogenesis of chronic radiation dermatitis, which is usually an irreversible condition; the main cytokines involved in inflammatory and fibrosis-stimulating processes are tumor necrosis factor-alpha (TNF-alpha), tumor growth factor-beta (TGF-beta), interleukins 6 and 1 (IL-6 and IL-1), connective tissue growth factor and platelet-derived growth factor (PDGF) [18].

Chronic radiation dermatitis is characterized by changes in the number of cells, fibrous tissue, pigmentation, and vascularity, clinically manifesting with hyperkeratosis, skin atrophy, cutaneous hyper or hypopigmentation, loss of hair follicles, sebaceous and sweat glands. The dilation of blood vessels due to irradiation leads to the appearance of telangiectasias. Furthermore, the damage to the blood vessels causes impaired circulation and cellular oxygenation with a consequent risk of ulceration, wounds, and skin necrosis [15].

Several factors influence the incidence and the severity of chronic radiation dermatitis, such as the total dose of radiations (+50 Gy to the skin), large treatment areas, altered fractionation, kind of radiotherapy, concurrent chemotherapy or targeted therapy, connective tissue or skin disorders, and genetic factors [11].

Anticancer therapies that can increase radiation sensitivity include EGFR and BRAF inhibitors, dactinomycin, doxorubicin, methotrexate, 5-fluorouracil, hydroxyurea, bleomycin, and tamoxifen.

Adopting preventive measures against radiodermatitis in patients receiving radiotherapy is essential; it is recommended to maintain proper skin hygiene, protect the skin from traumas, not wear tight clothes, and avoid sun exposure and high temperatures. The use of topical corticosteroids in the prevention of radiodermatitis remains controversial [11]. Emollients and moisturizers are recommended both as prophylaxis and for the treatment of erythema and dry desquamation. Intake of antioxidants, including vitamin E, vitamin C, selenium, and melatonin, can reduce radiation-induced cell damage [11,19]. Treatment varies according to the degree of severity of the radiodermatitis: moisturizers and low to medium potency topical steroids in case of dry desquamation and itching and hydrogel and hydrocolloid dressings for moist desquamation; skin necrosis and ulceration require discontinuation of radiotherapy, debridement, and usually a surgical intervention [15]. The use of pulse dye laser and intense pulsed light is effective in treating radiation-induced telangiectasias. Pulsed dye laser is effective and safe in patients with radiation-induced telangiectasias and atrophy, improving skin thickness, thanks to the remodeling effect of this laser procedure [10,20]. Management of ulcers and necrosis consists of hydrogel or hydrocolloid dressings, antibacterial agents, debridement, and surgical intervention for nonhealing ulcers and suspect lesions [15]. Low-intensity laser therapy has proven effective in increasing the number of blood vessels in chronic radiation ulceration [21].

### 4.2. Fibrosis

Fibrosis is a late effect of ionizing radiation therapy, which significantly reduces patients’ quality of life. Radiation-induced fibrosis (RIF) may occur 4 to 12 months after radiation therapy in the skin, subcutaneous tissue, and other organs exposed to irradiation (radiation dose > 50 gray) [22].

RIF’s pathogenetic mechanism is similar to the wound healing process; ionizing radiations cause DNA damage and induce reactive oxygen and nitrogen species production, stimulating inflammatory and fibrotic processes. Fibroblasts and myofibroblasts produce collagen, fibronectin, and proteoglycans resulting in increased tissue thickening, reduced tissue compliance, and functional alteration [22]. The transforming growth factor-beta (TGFβ) is the prevalent growth factor that mediates the fibrotic response.

Clinical presentation of RIF of the skin can be characterized by cutaneous induration and retraction, lymphedema, restricted movements, necrosis, and ulceration. Topical and oral medications, physical therapies are the current treatment options for RIF. Mechanical massage of the affected area can effectively counter tissue fibrosis, reducing pain, itching, and thickening of the skin. Oral administration of antioxidants such as alpha-tocopherol (vitamin E) can help protect cells from radiation-induced DNA damage. Similarly, pentoxifylline, a hemorheological agent, can effectively inhibit the proliferation of fibroblasts [23]. Other therapeutic agents studied for RIF treatment include hyperbaric oxygen therapy, superoxide dismutase (SOD), IFNγ, laser therapy with epidermal grafting [15].

### 4.3. Radiation Recall Dermatitis

Radiation recall dermatitis is a poorly understood and uncommon phenomenon characterized by the appearance of an acute inflammatory reaction in the area previously exposed to radiation, triggered by the administration of a chemotherapy agent or other medication [24]. Radiation recall dermatitis affects approximately 6% of patients undergoing RT, and in the majority of cases occurs when the drug is given months or years after irradiation. This condition is generally associated with a radiation dosage >20 gray and in some cases below 20 gray. The pathogenesis of this cutaneous phenomenon is unknown, but various theories have been proposed, including hypotheses regarding changes in stem cells in the irradiated area, cellular DNA damage and oxidative stress, and idiosyncratic drug hypersensitivity reactions [25].

Typical clinical manifestations of radiation recall dermatitis generally arise weeks after exposure to the trigger drug, sometimes during or immediately following intravenous administration. Clinically, the manifestations of radiation recall dermatitis vary according to the degree of severity and may present with a mild rash, dry desquamation, pruritus, swelling, edema, blistering, maculopapular rashes, ulceration, and skin necrosis may occur in severe cases [26].

Chemotherapeutic agents belonging to various classes may be responsible for triggering this skin reaction, including taxanes, alkylating agents, anthracyclines, antimetabolites, vinca alkaloids, EGFR inhibitors, BRAF tyrosine kinase inhibitors. It is unclear whether the radiation recall dermatitis is related to the regimen or the dosage of the chemotherapeutic agent. Moreover, non-chemotherapeutic agents can be implicated in the onset of radiation recall dermatitis, such as antimicrobial/antibacterial agents, non-steroidal anti-inflammatory agents, lipid-lowering agents, antitubercular drugs [25,27]. A case of radiation recall dermatitis induced by acyclovir has been reported in a woman who received radiotherapy for an intraductal carcinoma in situ [28].

The responsible drug must be delayed or stopped to allow for the resolution of manifestations. Topical or systemic steroids, non-steroidal anti-inflammatory drugs, and antihistamines can be used to relieve symptoms [26]. Mild reactions can undergo self-resolution.

### 4.4. Radiation-Induced Skin Malignancies

The role of radiation in promoting the development of skin cancers has been known for some time; in 1902, 7 years after the discovery of X-rays, Frieben reported this association for the first time. Basal cell carcinoma (BCC) represents the most frequent radiation-induced skin tumor [29]. BCC is characterized by slow growth and rarely metastasizes; there are several clinicopathological subtypes of this skin cancer: superficial, nodular, micronodular, morpheaform, infiltrative, and fibroepithelioma of Pinkus [30]. Treatment modalities for this type of skin cancer depend on the location, size, and type of lesion. Various therapeutic options include standard surgical excision, Mohs micrographic surgery, radiation, electrodesiccation and curettage, photodynamic therapy, cryosurgery, topical therapies, and systemic medications such as Vismodegib. Treatment of BCC is usually surgical, but some subtypes of BCC can be treated in different ways [30]. In 85% of cases, the radiation-induced BCCs are histologically nodular, followed by the superficial BCCs and, more rarely, fibroepithelioma of Pinkus and keloidal BCCs [31,32]. Generally, the latency period of BCCs can range from 2 to 70 years and after a radiation dose of up to 30 Gy [33,34]. The main risk factors for radiation-induced BCC are additional sun exposure, sun-sensitive skin, radiation exposure before age 20, ethnicity, genetic factors [29].

Several cases of radiation-induced squamous cell carcinomas (SCC) have also been reported [35]. Unlike BCCs, SCCs are more associated with metastasis; the treatment of choice is surgical excision [36,37].

The latent period for the onset of SCC after radiotherapy is approximately 20 years. Radiodermatitis often precedes the diagnosis of radiation-induced SCC [38]. Radiation-induced NMSCs tend to be more aggressive than those related to ultraviolet radiation and usually have poorly defined margins. A study by Edward et al. showed that radiation-induced SCCs were associated with a 5-year survival of 50%, compared with 90% for ultraviolet-associated SCCs [39].

Radiation-induced vascular proliferation, including atypical vascular lesions and cutaneous angiosarcomas, have also been reported. Angiosarcomas have been described after radiotherapy for carcinomas of the breast, ovary, cervix, vulva, endometrium, foreskin, non-Hodgkin’s lymphomas, and benign conditions [40]. Cutaneous angiosarcomas secondary to radiations are aggressive vascular tumors that generally occur 5 to 6 years after radiation therapy (radiation dose > 30 gray) and are characterized by poor prognosis; mean survival time after diagnosis ranges from 10.8 to 33.5 months [41]. These malignancies present as red/purple plaques or nodules with an ecchymotic appearance. Treatment involves a large surgical excision, given the high rate of local recurrences. Several cases of radiation-induced angiosarcomas in patients treated with radiotherapy for breast cancer have been described in the literature, with a median age of onset of 69 years and a latency period of 6 years [42]. Early diagnosis is fundamental, as the most important prognostic factor is the size of the lesion at the time of diagnosis; long-term follow-up is essential as radiation-induced angiosarcomas can occur beyond the 5-years oncological follow-up [43]. Angiosarcomas must be differentiated from atypical vascular lesions (AVL), also called lymphangiomatous papules, lymphangiomas, or acquired lymphangiectasias, which are benign vascular proliferations caused by lymphatic obstruction from surgery or radiation that can occur 3 to 4 years after radiation therapy (radiation dose 40–60 gray) [44]. AVLs may appear clinically as red or bluish papules or, in some cases, vesicles that are usually <5 mm in diameter, unlike angiosarcomas which are typically larger (7.5 cm). A biopsy is indicated to allow histological diagnosis [45]. Rare cases of malignant transformation of the AVLs have been reported [46].

In addition to angiosarcomas, malignant fibrous histiocytoma, leiomyosarcoma, and fibrosarcoma are other radiation-induced soft tissue sarcomas. These malignancies are characterized by a median age of 58 years and a latency period of 11 years [47]. Treatment for radiation-induced sarcomas includes surgery or chemotherapy. Osman Ali et al. reported a case of a 19-year-old female who developed four histopathologically different skin tumors following craniospinal irradiation for medulloblastoma: malignant epithelial adnexal tumor, glomangioma, Kaposiform hemangioendothelioma, and dermatofibrosarcoma protruberans [48]. Microcystic adnexal carcinoma and eccrine porocarcinoma, rare malignant cutaneous neoplasms of the sweat glands, have been reported in patients who received therapeutic irradiation [49,50]. Kaposi’s sarcoma, Merkel cell carcinoma, hamartoma, neoplastic lymphangitis, and melanoma have also been described as a late consequence of radiation therapy [29].

### 4.5. Morphea

Radiation-induced morphea (RIM) is a rare and often unrecognized complication associated with radiotherapy. Currently, no relationship with the radiation parameters has been highlighted. Morphea is an inflammatory condition characterized by the abnormal deposition of collagen in the dermis. Pathogenesis of RIM is unclear, but the literature data suggest that radiation exposure may stimulate cytokines production, such as interleukin 4, interleukin 5, and TGF-b, inducing the activation of fibroblasts, collagen synthesis, and consequent fibrosis. RIM differs from RIF, a common complication of radiations; RIM is characterized by a critical inflammatory infiltrate absent in fibrosis; moreover, RIF develops in previously irradiated areas, while RIM can also affect other skin areas [51]. More rarely, involvement of untreated skin areas may occur. Morphea that develops away from the previously treated area may be related to the development of neoantigens which causes T cells activation and release of TGF-β [52].

RIM is characterized by an inflammatory phase and a subsequent sclerotizing phase, also called “burn out” phase, in which fibrotic retraction and pigmentation occur [53]. Clinically, the lesions initially consist of erythematous and edematous plaques and subsequently become purplish and indurated, associated with pain and retraction.

A literature review analyzing 61 cases of RIM reported since 1989 highlighted that morphea frequently occurred in women who received radiotherapy for breast cancer; two cases of male patients have been reported [53]. RIM is often not diagnosed, as it is difficult to distinguish it from other clinically similar conditions such as radiation dermatitis, cancer recurrence, RIF. Therefore, it is essential to perform a biopsy and histological examination. The therapeutic management of RIM is not easy, as it can often be resistant to commonly used immunosuppressive agents, such as corticosteroids and calcineurin inhibitors. In mild cases, phototherapy (NB-UVB, UVA, UVA1) and topical treatment with tacrolimus, steroids with calcipotriol, and imiquimod are recommended [54].

Phototherapy and systemic treatment, including corticosteroids and methotrexate, should be considered in refractory cases [55].

### 4.6. Bullous Pemphigoid

Bullous pemphigoid (BP) is an autoimmune disease characterized by subepidermal blistering. BP is caused by autoantibodies directed to antigens of the hemidesmosome, BP180 or BPAG2, and BP230 or BPAG1e [56].

Radiation exposure is one of the many trigger factors of this bullous disease, along with medications, infections, thermal or electrical burns, transplants, surgical procedures, traumas, etc. [57]. BP may occur from the second week of radiation treatment to 16 years after, and it is associated with a minimum radiation dosage of 20 Gy [58].

It has been hypothesized that radiations may cause alterations of the antigenic properties of the cell surface, thus triggering the antigen-antibody reaction [59]. Another hypothesis is that low titer anti-basement membrane antibodies may already be in circulation in some cases, and subsequently, the radiation-induced tissue damage favors the deposition of these antibodies in the basement membrane [60]. Radiation-induced alterations of matrix metallopeptidase-9 (MMP-9) and growth factors including vascular endothelial growth factor receptor (VEGFR) levels have also been hypothesized [61]. Cases of radiation-induced BP localized only at the irradiated area, rarely at non-irradiated areas, and cases with generalized lesions have been reported in the literature [58]. Çalikoglu et al. presented a 94-year-old woman who initially developed BP localized to the irradiated area, which subsequently became generalized; the bullous eruption occurred four months after radiotherapy for breast cancer [62]. No clinical or histopathological differences between idiopathic and radiation-induced BP have been reported. Treatment is based on topical and systemic glucocorticoids, immunosuppressive drugs, systemic antibiotics, and anti-CD20 agents in refractory cases [58].

### 4.7. Lymphangioma Circumscriptum

Lymphangioma circumscriptum (LC) is a lymphatic malformation characterized by ectatic lymphatic channels in the dermis. Clinically it presents with clusters of translucent vesicles, while dermatoscopy shows yellow, reddish, and bluish lacunae with pale septa [63]. LC can be divided into congenital, occurring in infancy, and acquired, secondary to infections, invasive surgery, or RT in adults (radiation dosage 40–60 gray). The sites most frequently affected are the external genitalia, and more rarely, the upper limbs, axilla, and trunk. Treatment of radiation-induced LC is complex as relapses are very frequent; in many cases, treatment is not carried out due to the benign nature of the lesions. However, treatment modalities include laser therapy, sclerotherapy, surgical excision, cryotherapy, radiotherapy, and electrocautery [64]. Schulz et al. reported successful treatment with CO2 laser in a male patient who developed radiation-induced LC of the scrotum after a latency period of 8 years [65]. 

### 4.8. Pseudosclerodermatous Panniculitis

Pseudosclerodermatous Panniculitis (PIPP) is a rare complication of megavoltage radiotherapy. The first cases of PIPP were described in 1993 by Winkelmann et al. in four patients with breast cancer who received megavoltage X-ray photon [66]. It is an inflammatory condition characterized by the onset of indurated erythematous plaques localized in previously irradiated skin areas (radiation dosage > 50 gray). Histology shows lobular panniculitis with an inflammatory infiltrate with foamy histiocytes in the dermis and subcutaneous tissue, thickening and sclerosis of the septa, and adipocyte necrosis [67]. In some cases, other radiation-induced alterations may coexist, such as dilation and hyalinization of blood vessels, increase in fibroblasts and fibrosis, endothelial hyperplasia [66]. The latency period ranges from 1 month to 17 years after radiations. Several cases of post-radiation PPI have been reported in the literature, and most are female. Therefore, a role of hormones in this condition’s pathogenesis has been hypothesized; other pathogenetic hypotheses concern the release of profibrotic cytokines following radiation-induced damage, a delayed immune response to radiation-induced neoantigens, and gene polymorphisms related to TGF-β1 and retinoic acid [68].

The treatment consists of administering systemic steroids; PIPP can often undergo spontaneous resolution, so treatment is not always necessary [69].

### 4.9. Radiation Port Dermatophytosis

Radiation port dermatophytosis is the development of tinea corporis in the field of irradiation. The radiation dose associated with this condition ranges from 9 to 70.9 gray. Radiation port dermatophytosis was described for the first time by Maor in 1988 [70]. To date, few cases have been reported in the world literature [71]. Radiation port dermatophytosis can be underdiagnosed and mistaken for radiation-induced dermatitis. Moreover, in some cases, the two conditions can coexist.

Pathogenesis of radiation port dermatophytosis is unclear, and it may be influenced by several alterations induced by irradiation. Radiation may interfere with the function of precursor T-lymphocytes and Langerhans cells, reducing the immune response against fungal infections; furthermore, lymphedema and impaired lymph drainage can contribute to developing this condition [72]. The treatment consists of the administration of topical or systemic antifungals.

### 4.10. Others

Other skin conditions reported in the literature as a consequence of irradiation are: lichen planus [73], Langerhans cell histiocytosis [74], systemic mastocytosis [75], radiation port erythema multiforme [76]., solitary xanthogranuloma [77].

All radiation induced dermatological conditions are reported in Table 1.

## 5. Conclusions

Dermatologists and oncologists must know the full spectrum of skin complications associated with radiation treatment, especially given the high incidence in cancer patients. It is equally important to educate the patient about the preventive measures, such as adequate skin hygiene, photoprotection, the use of loose clothing, suitable moisturizers, and the importance of periodic skin self-control. The early diagnosis of the various dermatological conditions induced by radiation allows to establish an adequate therapy, reduce their evolution, and improve patients’ quality of life.

## Figures and Tables

**Table 1:** Main radiation-induced dermatological conditions.

Radiation-Induced Dermatologic Condition	Dose of Eposure	Pathogenesis	Clinical Manifestation	Treatment
**Acute radiation dermatitis**	≥2–40 Gy [11]	Damage to DNA, proteins, lipids and carbohydrates induced by free radicals; recruitment of inflammatory cells, apoptosis, and necrosis of epidermal cells [13,14].	Erythema to desquamation and ulceration [15]	Emollients, moisturizers, topical steroids, hydrogel and hydrocolloid dressing [15].
**Chronic radiation dermatitis**	>50 Gy [11]	Secretion of pro-inflammatory and profibrotic cytokines by inflammatory cells [18].	Teleangectasias, hyperkeratosis, atrophy, hyper or hypopigmentation, alopecia, ulceration, wounds, necrosis [9].	Lasertherapy, antibacterial agents, debridement, surgical intervention [10,15].
**Fibrosis**	>50 Gy [22]	Radiation induced DNA damage and reactive oxygen and nitrogen species production, stimulation of inflammatory and fibrotic processes [22].	Cutaneous induration and retraction, lymph-edema, restricted movements, necrosis and ulceration [45].	Mechanical massage, oral antioxidants, pentoxifylline, hyperbaric oxygen therapy, superoxide dismutase (SOD), IFNγ, laser therapy with epidermal grafting [15,23].
**Radiation Recall Dermatitis**	≥20 Gy in some cases below 20 Gy [25]	Changes in stem cells in the irradiated area, cellular DNA damage and oxidative stress, idiosyncratic drug hypersensitivity reactions [25].	Rash, dry desquamation, pruritus, swelling, edema, blistering, maculopapular rashes, ulceration and skin necrosis [26].	Topical or systemic steroids, non-steroidal anti-inflammatory drugs and antihistamines [25,26].
**Basal cell carcinoma (BCC)**	>30 Gy [29]	Direct DNA damage by ionizing radiation, and indirect damage caused by fee radicals; alteration in DNA repair processes, cell deasth, inflammation, angiogenesis [1].	The same as the BCCs of different etiology	Surgical excision, Mohs micrographic surgery, radiation, electrodesiccation and curettage, photodynamic therapy, cryosurgery, topical therapies, and systemic medication [29,31].
**Squamous cell carcinoma (SCC)**	>30 Gy [29]	=	The same as the SCCs of different etiology	Surgical excision [29].
**Cutaneous angiosarcomas**	>30 Gy [44]	=	Red/purple plaques or nodules with ecchimotic appearance [41].	Surgical excission [43].
**Atypical vascular lesions (AVL)**	40–60 Gy [44]	=	Red or bluish papules or vesicles.	Surgical excision [45].
**Morphea**	no relationship with the radiation parameters [51]	Abnormal secretion of cytokines causes activation of fibroblasts and an extensive fibrosis; development of neoantigens with T cells activation and release of TGF-β [51,52].	Eplaques which subsequently become purplish and indurated, associated with pain and retraction [53].	Phototherapy (NB-UVB, UVA, UVA1), tacrolimus, corticosteroids, calcipotriol, imiquimod, systemic corticosteroids [54].
**Bullous pemphigoid**	>20 Gy [58]	Alterations of the antigenic properties of the cell surface induced by radiation, trigger the anti-gen-antibody reaction [59]; radiation-induced tissue damage favors the deposition of these antibodies in the basement membrane [60].	Subepidermal blisters [59].	Topical and systemic glucocorticoids, immunosuppressive drugs, systemic antibiotics, anti-CD20 agents [58].
**Lymphangioma circumscriptum**	40–60 Gy [64]	Radiation-induced dilation and sequestration of lymphatic channels [63].	Clusters of translucent vesicles [63].	Lasertherapy, sclerotherapy, surgical excision, cryotherapy, radiotherapy and electrocautery [64].
**Pseudosclerodermatous Panniculitis.**	>50 Gy [67]	Release of profibrotic cytokines, delayed im-mune response to radiation-induced neoantigens, gene polymorphisms related to TGF-β1 and retinoic acid [68].	Indurated erythematous plaques [67].	Systemic steroids [69].
**Radiation port dermatophytosis**	9–70.9 Gy [70]	Alteration of the function of precursor T-lymphocytes and Langerhans cells; lymphedema and impaired lymph drainage [72].	Tinea corporis in the field of irradiation [70].	Topical or systemic antifungals [71].

## Data Availability

No new data was generated by this study.
